# Prof. A. H. Buchanan’s Introductory Address

**Published:** 1858-02

**Authors:** 


### Prof. A. H. Buchanan's Introductory Address.

We have received and read with pleasure the Introductory Address to the Medical Class of the Medical Department of the University of Nashville, for the present Session. It is an interesting and instructive address, and eminently characteristic of the man—scientific and practical—doing credit even to its learned and able author.

